# Familial Dysautonomia: Mechanisms and Models

**DOI:** 10.1590/1678-4685-GMB-2015-0335

**Published:** 2016-08-04

**Authors:** Paula Dietrich, Ioannis Dragatsis

**Affiliations:** 1 Department of Physiology, The University of Tennessee, Memphis, TN, USA

**Keywords:** Familial Dysautonomia, HSAN, IKAP, Ikbkap, ELP1

## Abstract

Hereditary Sensory and Autonomic Neuropathies (HSANs) compose a heterogeneous group of genetic disorders characterized by sensory and autonomic dysfunctions. Familial Dysautonomia (FD), also known as HSAN III, is an autosomal recessive disorder that affects 1/3,600 live births in the Ashkenazi Jewish population. The major features of the disease are already present at birth and are attributed to abnormal development and progressive degeneration of the sensory and autonomic nervous systems. Despite clinical interventions, the disease is inevitably fatal. FD is caused by a point mutation in intron 20 of the *IKBKAP* gene that results in severe reduction in expression of IKAP, its encoded protein. *In vitro* and *in vivo* studies have shown that IKAP is involved in multiple intracellular processes, and suggest that failed target innervation and/or impaired neurotrophic retrograde transport are the primary causes of neuronal cell death in FD. However, FD is far more complex, and appears to affect several other organs and systems in addition to the peripheral nervous system. With the recent generation of mouse models that recapitulate the molecular and pathological features of the disease, it is now possible to further investigate the mechanisms underlying different aspects of the disorder, and to test novel therapeutic strategies.

## Introduction

Hereditary Sensory and Autonomic Neuropathies (HSANs) compose a heterogeneous group of rare peripheral neuropathies characterized by loss of temperature and pain perception, in combination with other sensory and autonomic abnormalities. HSANs are classified clinically into five major types based on age of onset, mode of inheritance, and major clinical features (Dyck, 1993). Up to now, HSAN disease-causing mutations have been identified in over 10 genes. For instance, HSAN type IV, also known as Congenital Insensitivity to Pain with Anhidrosis (CIPA), is caused by mutations in the nerve growth factor (NGF) receptor (TrkA/NTRK1), and HSAN type V is caused by mutations in NGF that either prevent its processing, secretion, or downstream signaling (Rotthier *et al.*, 2012; Capsoni, 2014; Indo, 2014).

Familial Dysautonomia (FD, MIM 223900), also known as "Riley-Day syndrome", or HSAN type III, is the most prevalent HSAN. FD is an autosomal recessive congenital sensory and autonomic neuropathy that affects almost exclusively individuals of Ashkenazi, or Eastern European, Jewish extraction, although non-Jewish cases have also been reported (Guzzetta *et al.*, 1986; Leyne *et al.*, 2003; Silveira *et al.*, 2012; MIM 223900). FD was first described in 1949 (Riley *et al.*, 1949), based on reports of five children, all Jewish, who presented with diminished production of tears, excessive sweating and salivation, red blotching of the skin, reduced deep tendon reflexes, and marked arterial hypertension. Furthermore, two of the children did not complain when their feet were immersed in ice-cold water, suggesting impaired temperature perception. Over the following decade, additional cases with marked similarities were described, all of them of children with Jewish parents, suggesting that the disease was genetically inherited. The findings in the first few FD patients initially pointed to a central disturbance of autonomic function. Further investigations led to the recognition that several of the FD features, such as reduced pain and temperature perception, and cardiovascular abnormalities were caused by peripheral autonomic and sensory deficits. Despite marked similarities with other HSANs, FD has unique features that distinguish it from the other hereditary neuropathies. The clinical diagnosis of FD is based on the presence of the following cardinal features: absence of fungiform papillae on the tongue, absence of axon flare after intradermal histamine injection, decreased or absent deep tendon reflexes, absence of overflow emotional tears, and Ashkenazi Jewish descent (Axelrod and Pearson, 1984). Several symptoms of the disease are already present at birth, and worsen over time, suggesting that FD is a congenital progressive disorder. Initially, FD patients did not survive past childhood, but today with early intervention and supportive treatment the mean age of the FD population is approximately 15 years of age, with a 50% chance of surviving up to 40 years of age (Axelrod *et al.*, 2002; Gold-von Simson and Axelrod, 2006). Still, current treatments for FD are highly invasive, far from optimal, and there is no cure for this devastating disease. The most common causes of death are acute aspiration, chronic pneumonia and sudden death during sleep (Axelrod *et al.*, 2002). With the identification of the genetic mutation that causes FD, the last decade has been one of great advances in terms of understanding the mechanisms underlying FD, and also for unraveling the essential roles of its mutated gene, *IKBKAP*, and its encoded protein, IKAP, in multiple biological processes.

## Genetics and epidemiology

FD affects 1:3,600 Ashkenazi Jewish live births, with United States and Israel each having about 33% of the existing total population (Gold-von Simson and Axelrod, 2006). Carrier frequency ranges from 1/27 to 1/32 in the general Ashkenazi Jewish population, with individuals from Polish descent having a higher carrier frequency of 1/18 (Blumenfeld *et al.*, 1999; Dong *et al.*, 2002; Lehavi *et al.*, 2003). While it became clear early on that FD was a hereditary recessive disorder, the genetic basis of FD was only identified in 2001. By studying 26 families with multiple affected members, the FD gene (originally called "DYS"), was mapped to chromosome 9q31. Haplotype analyses of 441 FD chromosomes revealed the presence of a major haplotype observed in 435 (98.6%) of the cases (Blumenfeld *et al.*, 1999), indicating that almost all FD carriers share a common ancestor. The remaining six chromosomes revealed the existence of three other haplotypes: minor haplotype 1, detected in two unrelated families, minor haplotype 2 present in three families, and minor haplotype 3 in only one family. Importantly, in all cases these haplotypes were observed in individuals that were compound heterozygotes for the major haplotype.

### FD major haplotype

**Figure 1:**
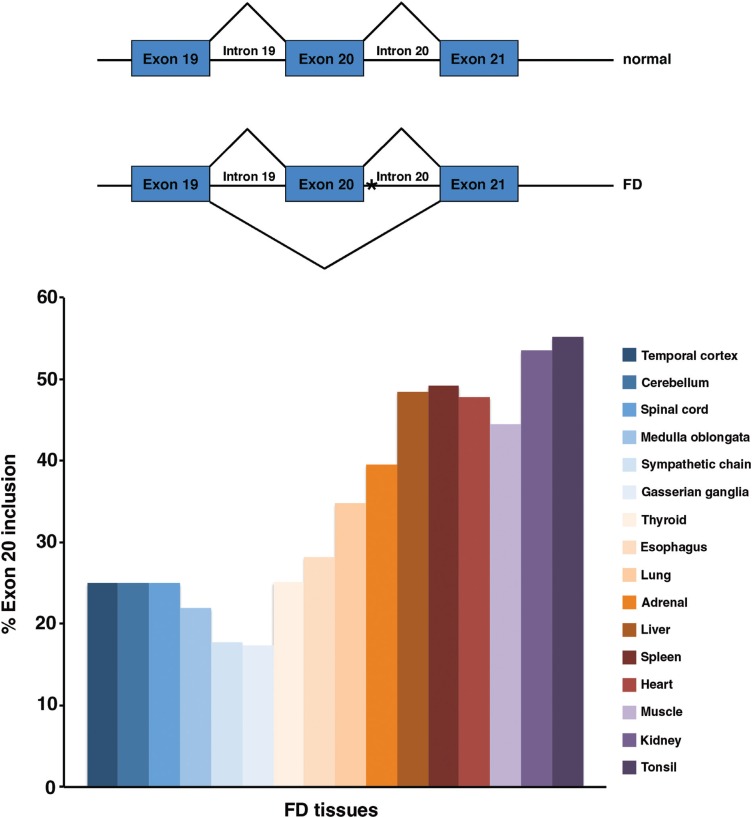
FD major haplotype mutation leads to tissue-specific exon 20 skipping in Ikbkap mRNA. (A) Schematic representation of Ikbkap normal and FD alleles. The FD major haplotype point mutation in position 6 of intron 20 is indicated by an asterisk (*). (B) Graphic representation of percentage of exon 20 inclusion in Ikbkap mRNA from FD tissues. (The graph represents a compilation and adaptation of the data presented by Cuajungco *et al.*, 2003 and Hims *et al.*, 2007). Note that neuronal tissues (represented in different shades of blue) consistently show severely reduced levels of exon 20 inclusion.

With the candidate gene mapped to a 471 kb interval (Blumenfeld *et al.*, 1999), two research groups independently screened for mutations by performing overlapping RT-PCR on mRNAs encoded by this region, using control and FD patient-derived lymphoblast and fibroblast cell lines. Primers to the transcript that encodes *IKBKAP* (inhibitor of kappa light polypeptide enhancer in B cells, kinase complex-associated protein, NCBI Reference Sequence: NM_003640.3, with mRNA length of 6129 bp) generated the predicted 218 bp RT-PCR product using mRNAs from control cells, and a 144 bp product when FD mRNA was used. Sequence comparison between the FD and control RT-PCR products revealed that the *IKBKAP* mRNA derived from FD cells does not contain exon 20. Sequence analysis of *IKBKAP* gene from FD chromosomes, revealed a T to C transition in position 6 of the donor splice-site of intron 20. While normal *IKBKAP* encodes a full-length protein (IKAP) of about 150 kDa, the FD mutation, (IVS20+6T > C), results in the generation of an mRNA in which exon 20 (74 bp) is spliced out, causing a frameshift that results in a premature stop codon (Anderson *et al.*, 2001; Slaugenhaupt *et al.*, 2001; IKAP, NCBI Reference Sequence: NP_003631.2, protein length 1332 aa). Significantly though, in FD patients there is variable tissue-specific skipping of exon 20, leading to reduced *IKBKAP* full-length mRNA levels in all tissues, with nervous system tissues displaying the most severe reduction in exon 20 inclusion (Figure 1; Cuajungco *et al.*, 2003; Hims *et al.*, 2007). Although the predicted truncated IKAP protein of 79 kDa has been detected in FD-derived lymphoblasts (Anderson *et al.*, 2001), other groups failed to detect such product in other FD cell lines or tissues. It is currently believed that at least part of the abnormally spliced *IKBKAP* transcripts is degraded by nonsense-mediated mRNA decay (NMD), as demonstrated in FD-derived olfactory stem cells (Boone *et al.*, 2010) and that the truncated protein might be unstable due to its inability to dimerize (Xu *et al.*, 2015).

To determine the underlying mechanism leading to alternative splicing and preferential skipping of exon 20 in neuronal tissues, an *in silico* analysis was performed to identify potential sequences that might also be involved in the process. Computational analyses showed that sequences 5' and 3' of exon 20, and the sequence of exon 20 itself provided an environment where the definition of exon 20 is weakened (Ibrahim *et al.*, 2007). In addition, differentiation of FD-derived induced pluripotent stem cells (iPSCs) into neuronal cells resulted in further reduction of exon 20 inclusion compared to non-differentiated cells, suggesting that the splicing machinery changes as the cells differentiate into neurons (Lee *et al.*, 2009).

### FD minor haplotypes

The most common minor haplotype (minor 2) (Blumenfeld *et al.*, 1999) is caused by a G to C transversion in exon 19 of the *IKBKAP* gene. The mutation results in both an arginine to proline substitution in aminoacid residue 696 of IKAP (R696P) and the disruption of a consensus serine/threonine kinase phosphorylation site, resulting in reduced phosphorylation at this site (Anderson *et al.*, 2001). FD minor haplotype 2 mutation is rare in the Ashkenazi Jewish population, with a carrier frequency estimated to be less than 1 in 2,500 and has only been detected in four patients, but never in homozygosity (Dong *et al.*, 2002). Minor haplotype 1 is caused by a C to T transition in exon 26 of the *IKBKAP* gene, resulting in a pro914-to-leu (P914L) substitution presumed to impair phosphorylation at this site. The mutation was identified in a patient of mixed ancestry, and was inherited from a non-Jewish mother (Leyne *et al.*, 2003). The phenotype caused by these mutations is currently unknown. The mutation responsible for minor haplotype 3 has not yet been characterized.

### Genetic testing and current trend of FD population

With the identification of the mutation responsible for most FD cases, carrier testing began. Carrier testing, based on DNA analyses, is available for the two most common mutations (major haplotype and minor haplotype 2), with an accuracy of 99%. In 2009, only five new FD cases were diagnosed in the world, representing a major reduction in new cases, considering that 15 or 20 were diagnosed per year in the 1990s (Couzin-Frankel, 2010).

Although the FD population at this moment is relatively small, there is still no cure for the existing patients, and the mechanisms underlying the disease are still poorly understood, precluding the development of more efficient therapies. In addition, since FD shares many similarities with other neuropathies, understanding the mechanisms of the disease may shed light into other related disorders and increase our understanding of normal peripheral and central nervous system development and maintenance.

## Clinical aspects and pathological findings

Although FD is genetically homogeneous and fully penetrant, clinical and pathological aspects of the disease are variable among individuals, and largely dependent on the age of the patient.

### Clinical Presentation

FD babies are born significantly smaller than their unaffected siblings with births weights ranging from 70 to 90% of the normal range, indicative of intrauterine growth retardation (Axelrod and Dancis, 1973). Poor suck and uncoordinated swallow is observed in 60% of FD infants, and, together with a high metabolism, can lead to failure to thrive and malnutrition. In fact, FD children and adolescents display very low fat content, even with high caloric intake. Difficulty swallowing may persist in older children, and appears to be the underlying cause of excessive drooling, a prominent feature of FD patients (Margulies *et al.*, 1968, Wolff *et al.*, 2002). Along with dysphagia, FD patients also display dysarthria due to oral incoordination (Gold-von-Simson and Axelrod, 2006). FD infants are typically hypotonic, and the average age for independent walking is about 26 months of age. Other gross milestones, including sitting unsupported, rolling over, and jumping are also significantly delayed. Decreased deep tendon reflex is also a typical characteristic in FD patients. Several of these features, including poor suck and uncoordinated swallow, and dysphagia have been attributed to either brainstem dysfunction or reduced muscle innervation and control. Hypothalamic neuroendocrine dysfunction has been hypothesized as a possible explanation for poor weight gain.

Although most FD patients appear to have normal intelligence, in general they tend to be literal and have difficulty extrapolating, and visual intellect often exceeds verbal abilities (Welton *et al.*, 1979). About 30% of FD children have signs of attention deficit disorder (ADD) characterized by short attention span and easy distractibility. Emotional lability is also among the prominent features of FD, and intensifies during episodes of crisis (see below). All these characteristics suggest central nervous system impairment.

FD patients share an unusual facial appearance, described as "trigonal face" with facial asymmetry (Riley *et al.*, 1949; Axelrod and Pearson, 1984). Cephalometric measurements showed that in FD patients the maxilla and mandible are retrognathe to the cranial base, a feature that is significantly more pronounced in the mandible. In addition, horizontal mandibular growth is also distinctive in FD. Together, these alterations give the impression of small jaws, and may contribute to difficulties in oral coordination and speech (Mass *et al.*, 1998). It has been suggested that chronic progressive denervation leading to differences in coordinated muscle function might be the primary cause of abnormal facial expression.

The most common orthopedic manifestation in FD is spinal deformity, with a prevalence of 48% at the age of 10, and 86% at the age of 15. About 50% of the spinal deformities are scoliosis only, 44% are kyphoscoliosis, and the remaining 4% kyphosis only (Kaplan *et al.*, 1997; Bar-on *et al.*, 2000; Laplaza *et al.*, 2001). Charcot joints and foot deformities (including equinovarus and cavovarus) develop during childhood in about 10-15% of FD patients (Bar-on *et al.*, 2000). In addition, FD patients sustain a much higher incidence of fractures compared to the normal age-matched population (63% versus 34%, respectively), most of them occurring before the time of skeletal maturation. Intriguingly, the number of fractures per individual is also higher than in the general population, despite the fact that FD children and adolescents are significantly less active than normal age-matched individuals. Progressive denervation is believed to be the cause of spinal deformity, while reduced pain perception is generally thought to be the underlying reason for increased fractures.

Ataxic gait is also one of the hallmarks of FD. FD patients adopt a wide stance, and exhibit unsteady walking, often requiring assistance to prevent falling when turning. The ataxia progressively worsens over time: by the age of 20, about 5% of the patients require walking aids, and the need progresses linearly so that by the age of 40, about 30% of the patients require assistance for walking (Bar-On *et al.*, 2000; Macefield *et al.*, 2011). Cerebellar dysfunction or loss of muscle spindle sensory afferents leading to reduced proprioceptive acuity have been proposed as possible underlying causes of ataxic gait (Anderson *et al.*, 2001; Macefield *et al.*, 2013).

A smooth tongue is observed in virtually all FD patients and represents one of the cardinal features of FD. In infants, the number and size of fungiform papillae on the tongue are already reduced (Pearson *et al.*, 1970), and fungiform papillae cannot be observed by the naked eye in older patients (Riley and Moore, 1966). The absence of filiform papillae, the reduced number and rudimentary development of fungiform papillae, together with the absence of taste buds, give the tongue a smooth appearance (Goldberg *et al.*, 1968; Pearson *et al.*, 1970).

In FD, decreased temperature and pain perception results in unrecognized burns and injuries. Temperature and pain perception are already impaired in early childhood in most FD patients, but marked variability is observed between individuals of the same age group. In a study involving 75 FD patients, mild temperature perception impairment was observed in about 20% of the patients examined, and severe impairment in about 47% of the cases. No significant differences were noted between young and older individuals and temperature perception did not worsen in a five-year follow-up. In the same group of patients, pain perception was normal in 20% of the young patients, and severely impaired in about 12% of the remaining patients of this age-group. Significantly, all older patients exhibited impaired pain perception, with severe impairment observed in more than 30% of the cases of this age group; in addition, in a five-year follow-up there was also a tendency for worsening (Axelrod *et al.*, 1981). Together, these observations indicate that the increased threshold for temperature perception does not change over time in FD patients, while there is a marked progression in impairment of pain perception. The decrease in pain and temperature perception are indicative of sensory deficits.

Ophthalmologic problems are also observed in FD and become worse as the patient population age. One of the main characteristics of FD is the absence of overflow emotional tears, and alacrima that may lead to corneal abrasions. Absent corneal reflexes, and abnormal pupillary response to metacholine are also typical findings (Axelrod, 2006). In addition, progressive optic nerve atrophy and visual decline are observed in a large fraction of FD patients, starting at the end of the first decade of life. In some patients, visual acuity and color vision deteriorate over time, likely due to progression of optic nerve damage (Rizzo 3rd *et al.*, 1986; Groom *et al.*, 1997; Mendoza-Santiesteban *et al.*, 2012; Mendoza-Santiesteban *et al.*, 2014).

Cardiovascular abnormalities are also prominent in FD patients. Orthostatic hypotension often starts in school-age children and become more pronounced with increasing age, in part due to the increase in height. In FD patients, postural hypotension, characterized by a severe decrease in blood pressure without compensatory increase in heart rate, results in episodes of lightheadedness, dizziness and weakness of the legs (Axelrod, 2004). Supine hypertension is also common in adolescents and becomes more frequent in older patients. FD patients are hypertensive (SBP > 140mmHg) about 20-50% of the time (Norcliffe-Kaufmann *et al.*, 2010; Carroll *et al.*, 2012) and older FD patients have a high incidence of left ventricular atrophy, a sign of end-organ target damage due to chronic hypertension. Renal function also deteriorates as the patients get older, with 20% of adult patients having reduced renal function. The severity of renal disease parallels the extent of blood pressure variability, and most likely happens as a secondary consequence of chronic hypertension (Norcliffe-Kaufmann *et al.*, 2013). The mechanisms leading to blood pressure variability and hypertension in FD have been sequentially attributed to central autonomic dysfunction (Solitare, 1991; Gold-von Simson and Axelrod, 2006), to sympathetic and parasympathetic efferent baroreflex dysfunction (Hilz *et al.*, 1999; Stemper *et al.*, 2004; Goldstein *et al.*, 2008), or, more recently, to afferent baroreflex failure due to vagal withdrawal (Norcliffe-Kaufmann *et al.*, 2010; Carroll *et al.*, 2012).

Vomiting attacks (gastroesophageal reflux), often associated with hypertension, tachycardia, diffuse sweating, and red blotching of the skin consist the most prominent manifestation of FD, occurring in about 65-80% of FD patients, and can occur either intermittently as a response to physical or emotional stress, or daily in response to the stress of arousal. This "constellation of signs" is commonly referred to as "dysautonomic crisis" and is the leading cause of frequent hospitalizations of FD children (Axelrod, 2004; Gold-von-Simson and Axelrod, 2006). Although the pathophysiology of FD crisis is still not fully understood, it appears to involve central autonomic dysfunction (Anderson and Rubin, 2005; Cheishvili *et al.*, 2014b)

Pulmonary problems are also common in FD patients. Aspiration due to misdirected swallow is the major cause of pneumonia in FD. If gastroesophageal reflux is also present, the risk for aspiration increases significantly and repeated pulmonary aspiration eventually leads to pulmonary disease (Gold-von-Simson and Axelrod, 2006). Breath-holding episodes, usually triggered by excitement, are also frequent, occurring in about 63% of FD patients, and can happen from 18 months of age up to 6 years of age. Prolonged breath-holding can be severe, due to insensitivity to hypoxia, and can lead to secondary complications. These episodes are thought to represent a type of seizure activity (Axelrod, 2004; Gold-von-Simson and Axelrod, 2006).

### Pathological, physiological and molecular findings

**Figure 2:**
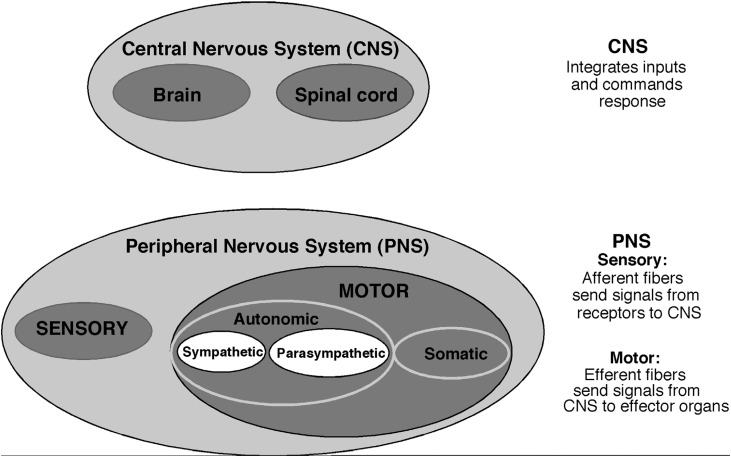
Simplified schematic representation of the Nervous System. The Central Nervous System (CNS), consists of brain and spinal cord, receives inputs from the Peripheral Nervous System (PNS) afferent (sensory) fibers, integrates the signals and commands the response via the efferent (motor) fibers of the PNS.

As mentioned above, several of FD major features could potentially be explained by central nervous system and/or peripheral nervous system abnormalities. Most of the pathological and physiological studies on FD have therefore focused on these two systems (Figure 2).

#### Central nervous system

In initial reports dating from 1945 to 1964, central nervous system (CNS) pathology was described in four FD patients ranging from 2 to 18 years of age. In all four cases, demyelination of the reticular formation of the pons and medulla were consistent findings. In addition, demyelination of spinothalamic tracts, and loss of cranial nerve nuclei of the brain stem were also reported in two cases (Aring and Engel, 1945; Cohen and Solomon, 1955; Brown *et al.*, 1964). Other abnormalities, including thalamic degeneration and brain abscesses were observed in only two cases, but appeared to be secondary to episodes of hypoxia or inflammation (Solitare, 1991). However, subsequent investigations in an additional four FD patients (ranging from 10 months of age to 31 years of age) failed to detect any signs of demyelination or other signs of neuronal loss or atrophy in the CNS (Pearson *et al.*, 1970; Yatsu and Zussman, 1964; Solitare and Cohen, 1965; Fogelson *et al.*, 1967).

Due to the apparent inconsistency of CNS findings in FD patients, and the concomitant realization that not only several of the FD features could be explained by peripheral sensory and autonomic disturbances, but also that peripheral nervous system appeared more consistently altered in FD (see below), subsequent studies focused almost exclusively on the evaluation of peripheral nervous system anomalies in FD patients, with a nearly complete disregard to CNS evaluation for almost 40 years.

More recently however, additional studies have provided a strong indication that the CNS is also compromised in FD. For instance, visual impairment in FD patients become noticeable after the first decade of life, and are associated with progressive optic atrophy and with predominant loss of papillomacular nerve fibers (Rizzo 3rd *et al.*, 1986; Groom *et al.*, 1997; Mendoza-Santiesteban *et al.*, 2012, 2014). Analyses of brainstem auditory evoked potentials, as well as blink and jaw jerk reflex in FD patients also strongly suggest brainstem dysfunction (Lahat *et al.*, 1992; Gutierrez *et al.*, 2015; Tellez, 2015). MRI studies showed abnormalities that suggest compromised myelination as well as grey matter and white matter micro-structural damage in FD brains. Abnormal findings are more evident in optic radiation, middle cerebellar pedunculum, and frontal lobe (Axelrod *et al.*, 2010). Significantly, analyses of FD brains showed that the levels of several transcripts and their respective proteins that are involved in myelination are severely reduced in FD brains compared to age-matched healthy individuals (Cheishvili *et al.*, 2007). These observations indicate that central nervous system abnormalities indeed contribute to FD clinical findings.

#### Peripheral nervous system

##### Autonomic nervous system

In one of the first reports, degenerative pigmentary changes and vacuolization in the cytoplasm were observed in thoracic and pelvic sympathetic ganglia of two young FD siblings, ages 2 and 8 (Brown *et al.*, 1964). Subsequent work in three additional young FD infants (ranging from 10 months to 2 years of age) confirmed these initial findings (Solitare and Cohen, 1965; Freytag and Lindenberg, 1967; Pearson *et al.*, 1970). Post-mortem analyses performed in two of these infants revealed that in the cervical and thoracic sympathetic ganglia several neurons were small, and overall neuronal numbers were reduced, estimated to be around 30% of normal (Freytag and Lindenberg, 1967; Pearson *et al.*, 1970; Solitare, 1991). Degenerative changes with signs of inflammation were also observed (Pearson *et al.*, 1970). Quantitative analyses performed in adult FD patients revealed that the mean volume of superior cervical ganglia (SCG) is reduced to 34% of the normal range, neuronal density to 37% of normal, with neuronal numbers as low as 10% of controls, indicating progressive loss of neuronal cells with age (Pearson and Pytel, 1978). Immunohistochemistry for tyrosine hydroxylase (a rate-limiting enzyme of catecholamine biosynthesis) demonstrated that although there was a severe depletion in SCG neurons, the remaining neurons expressed higher levels of tyrosine hydroxylase compared to controls, which has been interpreted as being part of a compensatory mechanism (Pearson *et al.*, 1979). These analyses clearly indicate that sympathetic neurons are reduced in numbers and are morphologically abnormal in FD already at early age, and it is currently assumed that intrauterine development of sympathetic ganglia is severely compromised in FD.

Consistent with the reduction in neuronal numbers in sympathetic ganglia, ultrastructural studies of peripheral blood vessels also demonstrated the absence of autonomic nerve terminals. In addition, catecholamine metabolism is altered in FD patients (Smith *et al.*, 1963), and other physiological studies indicate that sympathetic deficits also lead to cardiac hypo-innervation (Goldstein *et al.*, 2008).

In the parasympathetic system, the sphenopalatine ganglion is majorly compromised, with a significant reduction of neuronal numbers, averaging 20% of controls. The ciliary ganglion on the other hand displays only a mild reduction in total neuronal numbers (Pearson and Pytel, 1978).

Sympathetic and parasympathetic denervation and dysfunction are consistent with several of FD clinical features, including defective lacrimation, impaired pupillary reflex, impaired body temperature control, skin blotching, and excessive sweating. Hypertension and postural hypotension have also been attributed to sympathetic deficits, although this is currently under discussion.

##### Sensory nervous system

Neuronal numbers in dorsal root ganglia (DRG) are already diminished in young FD patients, and can be as low as 10-20% the numbers of normal age-matched individuals (Pearson *et al.*, 1978), although extensive variability has been observed between individuals (Fogelson *et al.*, 1967; Solitare, 1991). More consistently, demyelination of the posterior columns (dorsal columns) of the spinal cord has been demonstrated in most post-mortem analyses (Fogelson *et al.*, 1967; Pearson *et al.*, 1978; Solitare, 1991). The finding of severe depletion of DRG neurons early postnatally is suggestive of impaired development during embryogenesis. However, degenerative changes in sensory ganglia (nodules of Nageotte) have been demonstrated in at least some of the cases, suggesting that progressive degeneration also occurs postnatally (Pearson *et al.*, 1970, 1978).

Biopsies from the back and calf of FD patients revealed significant loss of nerve fibers in the epidermis and subepidermal neural plexus, with epidermal nerve fibers (ENF) densities averaging about 12-15% that of controls. In particular, sensory (substance P (SP) and calcitonin gene-related peptide (CGRP) positive) nerve fibers were virtually absent in FD biopsies, indicating significant loss of sensory skin innervation. In contrast, vasoactive intestinal peptide (VIP) staining, usually absent in normal subepidermal plexus, was significantly increased. In addition, a significant number of empty Schwann cell sheaths were also observed, indicating recent denervation (Hilz *et al.*, 2004). The presence of VIP staining and empty Schwann cell sheaths is indicative of inflammation, ongoing denervation, with some capability for regeneration, consistent with the clinical observation that FD is a progressive neurodegenerative disorder. The decreased unmyelinated nerve content with virtual absence of CGRP and SP immunostaining is compatible with the decrease in pain and temperature perception.

Sural nerve biopsies from FD patients exhibit a very consistent pattern that also distinguishes FD from all the other HSANs. Compared to normal individuals, the sural nerve of FD patients has diminished fascicular area (50% the area of controls), displays severe depletion of unmyelinated axons, and of small myelinated fibers. As observed in skin biopsies, there is also a significant number of empty Schwann cell sheaths and occasional presence of macrophages, indicative of ongoing axonal loss (Aguayo *et al.*, 1971; Pearson *et al.*, 1975; Guzzetta *et al.*, 1986).

Histopathological analyses from eight FD patients and age-matched controls demonstrated that in FD there is an extreme paucity in the geniculate ganglion neuronal numbers, as well as a significant reduction in neurons in the vestibular ganglion (cranial ganglion VIII), albeit to a lesser extent (Tokita *et al.*, 1978). Since the geniculate ganglion innervates the tongue fungiform papillae taste buds, this reduction in neuronal numbers is consistent with both the reduced sensory innervation and the absence of taste buds demonstrated in FD tongue (Pearson *et al.*, 1970), which require sensory innervation for their proper development and survival. Histopathological changes in the vestibular ganglion may in turn explain the poor balance and coordination of FD patients (Tokita *et al.*, 1978). So far, no histopathological analyses have been performed in other cranial sensory ganglia in FD.

Development of the peripheral nervous system begins with delamination of neural crest cells (NCC) from the neuroectoderm. These cells give rise to sympathetic ganglia and most of the sensory ganglia, including dorsal root ganglia (DRG) and trigeminal ganglia. In parallel, cells derived from the cranial placodes also delaminate and will contribute to the cranial sensory ganglia, geniculate, petrosal and nodose ganglia (Steventon *et al.*, 2014). Cells derived from these two origins then either coalesce or migrate to their respective final locations where they undergo differentiation into several neuronal subtypes. Virtually all neurons of the peripheral nervous system undergo programmed cell death shortly after the time of initiating functional innervation. Survival of neurons during the period of innervation depends on neurons acquiring access to a limited supply of neurotrophic factors expressed by the innervated "target field" and retrogradely transported to the neuronal cell body. For instance, the requirement of nerve growth factor (NGF) as a target field-derived survival factor for sympathetic and sensory nociceptive neurons is well established (Indo, 2014). In the developing rat, NGF expression levels are highest in tissues that are highly innervated by sympathetic fibers (Shelton and Reichardt, 1984), while sensory innervation correlates with NGF expression in the developing skin and spinal cord (Davies *et al.*, 1987; Elkabes *et al.*, 1994). Similarly, other types of neurons require appropriate supply and signaling by other neurotrophins. Neurotrophic support derived from the target field is also required throughout postnatal life for maintenance of innervation and neuronal survival. Hence, disturbance of any of these steps (or a combination of them) could result in depletion of sensory and/or sympathetic neurons and loss of innervation in FD.

## IKAP Cellular functions: implications for PNS deficits

Historically, IKAP was identified as a novel 150 kDa protein that interacts with cytokine-activated IκB kinase (IKK) complexes, which participate in the activation of the transcription factor NF-κB, hence the name IKAP, for "IKK complex associated protein". IKAP was initially shown to bind directly to IKK-alpha and IKK-beta and, acting as a scaffold protein, to assemble IKKs into an active kinase complex (Cohen *et al.*, 1998). Investigations by other research groups confirmed that IKAP is mostly localized in the cytoplasm, but failed to identify IKAP as a regular member of IKK complexes and further demonstrated that IKAP is not required for NF-κB signaling (Krappmann *et al.*, 2000). Since then, several other cellular functions of IKAP have been unraveled. IKAP, either as part of the Elongator complex (see below) or possibly acting independently, has been shown to interact with a variety of nuclear and cytoplasmic proteins and to play a role in tRNA wobble uridine modification (Lin *et al.*, 2013; Karlsborn *et al.*, 2014; Laguesse *et al.*, 2015), cytosolic stress signaling (Holmberg *et al.*, 2002), DNA repair (Li *et al.*, 2009), and zygotic paternal DNA demethylation (Okada *et al.*, 2010), among others. However, so far the link between any of these above-mentioned functions with FD pathology and in particular with PNS deficits still remain to be elucidated.

This section will therefore focus only on the cellular functions of IKAP that have been more thoroughly investigated and have led to insights into the mechanisms underlying abnormal PNS development and function in FD.

### IKAP is a member of the Elongator Complex

Sequence comparison between species showed that IKAP is the human homologue of the yeast elongator protein 1 (ELP1), one of the six subunits of the Elongator complex, which was initially identified as a complex essential for RNA polymerase II (RNA pol II) transcription elongation (Otero *et al.*, 1999). Purification and characterization of the human Elongator complex revealed that, as in yeast, the human holo-Elongator complex is also composed of six subunits, and confirmed that IKAP is an integral component of the core of the Elongator complex, which is composed of three subunits hELP1/IKAP, hELP2 and hELP3 (Hawkes *et al.*, 2002). Within the Elongator complex, ELP1/IKAP appears to act as a scaffold protein required for Elongator assembly and also serves to dock other proteins that regulate the Elongator function, whereas ELP3 is the catalytic subunit and acetylates histones H3 and H4 (Glatt and Muller, 2013). Consistent with these observations, IKAP protein is also observed in the nucleus, and decreased IKAP expression in FD patient-derived fibroblasts leads to reduced ELP3 levels, reduced Elongator binding, and decreased histone H3 acetylation in the coding region of genes that are downregulated in FD cells. Moreover, RNA pol II density is significantly decreased at the 3' end of these genes, but not at the promoter region, implying that the Elongator complex is not required for recruitment of RNA pol II to the promoter region, but affects primarily transcript elongation (Close *et al.*, 2006). These findings suggested that transcriptional dysregulation might be the underlying cause of the deficits observed in FD.

### Decreased IKAP expression impairs cell migration

With the finding that IKAP is part of the RNA pol II transcriptional Elongator complex, much of the subsequent work focused on comparative transcriptome or microarray analyses, aiming at identifying pathways that are affected by IKAP depletion. Using this approach, it was found that gene transcripts involved in cell migration are significantly down-regulated in a variety of FD patient-derived cells, including fibroblasts (Close *et al.*, 2006), induced pluripotent stem cells (iPSCs, Lee *et al.*, 2009), and olfactory ectomesenchymal stem cells (hOE-MSCs, Boone *et al.*, 2010), as well as in *IKBKAP* siRNA- transfected cell lines including HeLa (Close *et al.*, 2006), and SHSY5Y neuroblastoma cells (Cohen-Kupiec *et al.*, 2011).

Consistent with the down-regulation of transcripts involved in cell migration, cell migration defects were demonstrated *in vitro* for all of the above-mentioned FD-derived cell types. These observations reinforced the notion that FD peripheral nervous system abnormalities might be the result of impaired migration of neural crest cell (NCC) precursors. *In vivo* studies however, demonstrated that inactivation of the *IKBKAP* gene in NCC precursors, either in the chick or in the mouse, does not impact their migration. In the chick, down-regulation of IKAP expression in NCC by *Ikbkap* small-hairpin RNA (shRNA) or *Ikbkap* si-RNA microinjection or electroporation revealed that IKAP function is not necessary for either NCC migration or DRG formation (Hunnicutt *et al.*, 2012; Abashidze *et al.*, 2014). Similarly, conditional inactivation of the mouse *Ikbkap* gene in pre-migratory and migratory NCCs does not affect NCC migration, pathfinding, or formation of DRG and sympathetic ganglia (George *et al.*, 2013; Jackson *et al.*, 2014).

Together, these findings indicate that although reduction or loss of IKAP expression results in impaired cell migration, this impairment is either compensated by external signals *in vivo*, or are not severe enough to have a biological significance. In any case, it is clear that peripheral nervous system abnormalities in FD are not caused by impaired cell migration.

### IKAP and cytoskeleton organization: impact on neurite outgrowth and intracellular transport.

A much more consistent and biologically relevant cellular function of IKAP appears to be its requirement for cytoskeleton actin filament and microtubule organization and neurite outgrowth. Cytosolic IKAP was shown to co-purify with filamin A in rat cerebellar granule neurons, and immunohistochemistry for IKAP and filamin A showed that they co-localize with membrane ruffles. Depletion of IKAP in these cells did not decrease the expression of genes involved in cell migration, but resulted in actin cytoskeletal disorganization and reduced cell migration *in vitro* (Johansen *et al.*, 2008). These observations suggest that cell migration defects in cells depleted of IKAP may be linked to cytoskeleton disturbances instead of transcriptional dysregulation.

Immunostaining of FD-derived fibroblasts for the cytoskeleton component α-tubulin revealed disorganization of the microtubules (MTs) that resulted in aberrant cell shape. Similar results were obtained with neuroblastoma cells after down-regulation of IKAP. Moreover, in neuroblastoma cells, α-tubulin was shown to be concentrated in one pole of the cells resulting in abnormal process formation, with concomitant up-regulation of the MT-destabilizing protein SCG10 (STMN2). Significantly, RNAi-mediated SCG10 downregulation in FD-derived fibroblasts rescued cytoskeleton organization (Cheishvili *et al.*, 2011).

Microtubules (composed of alpha and beta-tubulin dimers) are particularly abundant in neurons, and are involved in multiple intracellular processes, including developing and maintaining cell shape, intracellular transport, cell signaling, and neurite extension (Kapitein and Hoogenraad, 2015). *In vivo* studies confirmed the need of IKAP for all these processes.

In the developing mouse brain, *Ikbkap* silencing in migrating projection neurons results in altered cell morphology and absence of growing apical dendritic tree and processes. In culture, *Ikbkap* null cortical neurons also exhibited reduced dendrite length and branch numbers (Creppe *et al.*, 2009). Interaction of IKAP with ELP3 appears to be necessary to regulate alpha-tubulin acetylation, a process that appears to be required for branching of postmigratory projection neurons (Creppe *et al.*, 2009; Nguyen *et al.*, 2010). Importantly, purified ELP3-enriched fraction promotes α-tubulin acetylation *invitro* suggesting a direct role of IKAP and ELP3 in this process (Creppe *et al.*, 2009).

Down-regulation of *Ikbkap* in chick neural crest cell precursors lead to marked disruptions in axonal projections of DRGs (Hunnicutt *et al.*, 2012; Abashidze *et al.*, 2014). In particular, the major disturbances are observed in the branching and positioning of the distal processes, while the positioning of the main nerves do not appear to be affected. In growth cones of control DRG cultures, IKAP was shown to co-localize with stable tubulin fibers, and to a lesser extent along dynamic tubulin fibers. Significantly, depletion of IKAP in cultured DRGs results in disturbance of tubulin structures (Abashidze *et al.*, 2014). Similarly, in mice, inactivation of *Ikbkap* gene in neural crest cells leads to 35 to 40% reduction in total neuronal numbers in DRGs (George *et al.*, 2013; Jackson *et al.*, 2014), and reduction in sympathetic neuronal numbers between 42% loss (Jackson *et al.*, 2014) to 70% loss (George *et al.*, 2013). Similar to chicks, loss of IKAP in neural crest cells in mice leads to a significant reduction in sympathetic and sensory target tissue innervation, likely due to abnormal axonal branching (Jackson *et al.*, 2014).

Together these results indicate that IKAP is required for axonal branching and fine-tuning of the innervation process, and suggest that loss of DRG and sympathetic neuronal cells in FD is likely a consequence of failed innervation of target tissues.

Recently, it has also been shown that downregulation of IKAP in cultured chick DRG neurons results in decreased NGF intracellular signaling. Moreover, IKAP was also shown to interact directly with the motor protein dynein, suggesting that IKAP may also be involved in intracellular transport (Abashidze *et al.*, 2014). These initial findings were further corroborated by transcriptome analyses in neuronal differentiated FD-derived embryonic stem cells as well as in FD embryonic brains (Lefler *et al.*, 2015). In particular, these analyses revealed that in FD-derived neurons and embryonic brains synaptic vesicular and neuronal transport genes are directly or indirectly affected by IKAP depletion (Lefler *et al.*, 2015). Significantly, among the five types of HSANs, FD (HSAN III), HSAN IV (caused by mutations in NGF receptor), and HSAN V (caused by mutations in NGF) share several similarities. All these disorders are congenital and are characterized by decreased pain and temperature perception, Charcot joints, decreased skin innervation, and severe depletion of unmyelinated fibers and small myelinated fibers in the sural nerve (Capsoni, 2014). Null mutations for NGF or NGF receptor (TrkA/NTRK1) in mice result in severe loss of sympathetic neurons and nociceptive sensory neurons (Davies, 2000).

Together, these observations suggest that IKAP may play an essential intracellular role in retrograde neurotrophic transport and signaling, and that in addition to impaired target innervation, impaired neurotrophic signaling might also contribute to PNS neuronal cell death in FD.

## What does expression of IKAP in the developing embryo tell about FD?

Up to now, there is limited information regarding the pattern of expression of IKAP during embryogenesis, and although FD appears to be a developmental disorder, it is currently not known what is the tissue-specific pattern of exon-20 skipping during development, since all the information regarding alternative splicing in FD has been performed in adult-derived tissues and cell lines (see above).

*Ikbkap* mRNA is already detected in early mouse embryos (embryonic day 8.5; E8.5), prior to the formation of NCCs, indicating that IKAP may be involved in other developmental processes as well (Chen *et al.*, 2009; Dietrich *et al.*, 2011). High levels of IKAP expression are later on observed in sensory and sympathetic neuroblasts and in the developing brain, as shown by immunohistochemistry (Creppe *et al.*, 2009; Jackson *et al.*, 2014). RT-PCR analyses also detected low-to-moderate levels of *Ikbkap* expression in peripheral organs, including heart, kidney, and lungs in developing mouse embryos at mid-to-late gestation (Dietrich and Dragatsis, unpublished results).

In the chick, analysis of the pattern of expression of IKAP was focused exclusively in the developing peripheral nervous system (Hunnicutt *et al.*, 2012; Abashidze *et al.*, 2014). *In situ* hybridization and immunohistochemistry failed to detect IKAP expression during the period of neural crest migration and DRG formation. Instead, IKAP expression is first observed in nascent and postmitotic neurons, and increases as the neurons mature and differentiate (Hunnicutt *et al.*, 2012; Abashidze *et al.*, 2014). In particular, the increase in IKAP expression in DRGs coincides with the onset of peripheral outgrowth and target innervation (Abashidze *et al.*, 2014). In addition, significant immunolabeling for IKAP was also observed in the developing spinal cord, in particular in the ventral horn motor neurons and the ventricular zone (Hunnicutt *et al.*, 2012), and in primary sympathetic ganglia (Abashidze *et al.*, 2014).

A far more detailed analysis has been performed in the developing rat. *In situ* hybridization in mid-to-late gestation rat embryos (E15.0 to E21.0) revealed that *Ikbkap* mRNA is expressed in a wide variety of tissues. At E15.0, *Ikbkap* mRNA is found in the brain, spinal cord, sensory ganglia, eye retina, liver, and intestinal tract. By E17.0, *Ikbkap* mRNA begins to be expressed in additional tissues, including the adrenal gland, Merkel cells, salivary glands, lungs, renal cortex, and cartilage. At E21, in addition to the tissues listed above, a high level of *Ikbkap* expression is also detected in the heart, skin, stomach, and carotid body. Notably, at all stages examined, the highest levels of expression were seen in the dorsal root ganglia, trigeminal ganglia, retina, central nervous system, and kidney cortex (Mezey *et al.*, 2003).

The wide distribution of *Ikbkap* mRNA expression observed in the developing rat implies that IKAP may play essential roles not only in nervous system tissues but also in non-neuronal cells. In this aspect, it is important to note that expression of IKAP in the kidney correlates well with the relatively high frequency of kidney malformations observed among FD patients, and suggests that a need of IKAP in the kidney may also contribute to kidney disease (Norcliffe-Kaufmann *et al.*, 2013). Expression of IKAP in the retina could explain the selective loss of retina ganglion cells (Mendoza-Santiesteban *et al.*, 2014). Likewise, expression of IKAP in cartilage is consistent with the decrease in bone mineral density observed in FD patients, a possible underlying explanation for the increased frequency of fractures (Maayan *et al.*, 2002), and suggests that IKAP may also be required for bone growth. Finally, and most intriguingly, the presence of *Ikbkap* mRNA in peripheral target tissues (salivary glands, skin, heart, lungs, stomach) suggests that IKAP may also play an essential role in these tissues. An attractive possibility is that IKAP might be required for adequate production of neurotrophins that are retrogradely transported to neuronal cells at the time of innervation as well as postnatally in order to maintain innervation. This possibility is substantiated by the histopathological changes in the skin of FD patients where there are signs of denervation and re-innervation, and also by the early biochemical findings that suggested that NGF produced by FD-derived fibroblasts cells demonstrates decreased neurotrophic activity (Siggers *et al.*, 1976; Schwartz and Breakefield, 1980; Wrathall, 1986).

## Animal models for FD

The pattern of IKAP expression and the phenotypic features of FD not related to the peripheral nervous system suggest that FD is a complex disease and could actually be viewed as a multiple system disorder. In order to fully understand the pathogenesis of FD and to develop more comprehensive and efficient therapies, animal models that faithfully recapitulate the disease would be greatly advantageous.

### Mouse Ikbkap

The mouse *Ikbkap* gene is located on chromosome 4 and encodes a 6160 nucleotide long mRNA (NCBI Reference Sequence: NM_026079.3). At the nucleotide level, the mouse Ikbkap mRNA exhibits 77% identity with the human IKBKAP mRNA (Coli *et al.*, 2001; Cuajungco *et al.*, 2001). As in humans, the mouse *Ikbkap* gene consists of 37 exons, distributed over about 53 Kb of genomic DNA. Importantly, the donor splice-site sequence altered in FD major haplotype is also conserved in the mouse. The high level of conservation of the gene between human and mouse suggest that the function of the gene is also conserved between species.

### Inactivation of Ikbkap gene in the mouse

**Figure 3:**
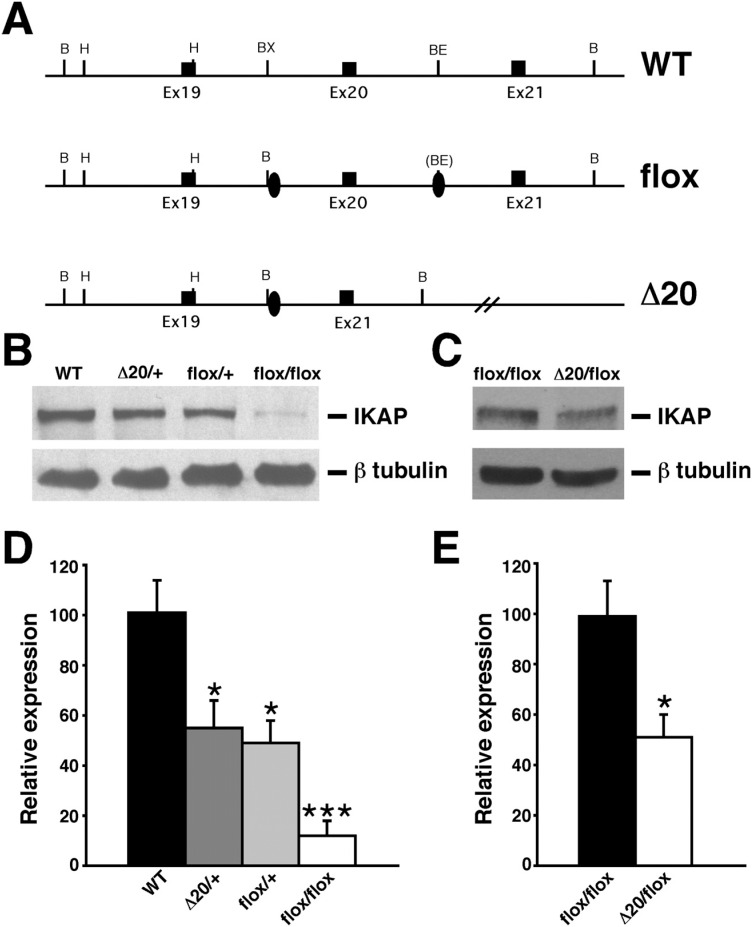
Generation and molecular characterization of FD mouse models. (A) Schematic representation of wild-type allele (WT), Ikbkap flox allele (flox), and Ikbkap allele lacking exon 20 (Δ20). Exons are represented by black rectangles, and black ovals represent the loxP sites. Restriction sites shown on the schematic are: Bam HI (B), HindIII (H), BstXI (BX) and BstEII (BE). (B) Western blot analyses of total protein lysates from forebrain of 11-month-old WT, *Ikbkap* ^Δ*20/+*^, *Ikbkap* ^*flox/+*^, and *Ikbkap* ^*flox/flox*^ mice. Upper panel shows detection of IKAP with the polyclonal anti-IKAP antibody (AnaSpec), and lower panel shows anti-β-tubulin staining for loading control. Note that IKAP protein level is severely reduced in *Ikbkap* ^*flox/flox*^ brain. (C) Western blot analyses of total protein lysates from whole brain of E16.5 *Ikbkap* ^*flox/flox*^ and *Ikbkap* ^Δ*20/flox*^ embryos. Upper panel shows detection of IKAP with the polyclonal anti-IKAP antibody (AnaSpec), and lower panel shows anti-β-tubulin staining for loading control. Note that in *Ikbkap* ^Δ*20/flox*^ brain IKAP protein expression is reduced compared to *Ikbkap* ^*flox/flox*^ brain. (D and E) Quantitative analyses of IKAP expression levels in mouse brains of different genotypes. (D) IKAP expression levels were normalized over tubulin levels and are expressed as percentage of WT. (E) IKAP expression levels were normalized over tubulin levels and are expressed as percentage of *Ikbkap* ^*flox/flox*^. Data are represented as Mean ± SD; n = 3 experiments. *P < 0.05, ***P < 0.001.

In an initial attempt to understand the roles of IKAP in embryogenesis and possibly generate a mouse model for the disease, two research groups generated null mutations in the mouse *Ikbkap* gene. Unexpectedly, it was found that inactivation of the *Ikbkap* gene in mice results in embryonic lethality, between E10.5 and E11.5 (Chen *et al.*, 2009; Dietrich *et al.*, 2011). *Ikbkap* null embryos appear grossly normal at E6.5 (Chen *et al.*, 2009), but by E9.5 they display already a significant developmental delay (about 24 hr delay) compared to controls. By E10.5, although mutant embryos reach several milestones of a typical E9.5 embryo (including neural fold closing, turning of the embryo, and development of the first branchial arch) they display poor vascularization of the extraembryonic tissues, anterior cephalic developmental defects, and cardiovascular abnormalities. In particular, development of the forebrain is compromised, and the heart does not develop past an E8.5-like primitive heart. Abnormal heart looping and dilated pericardial sac in null embryos indicate that heart failure is likely the cause of death of *Ikbkap* null embryos (Dietrich *et al.*, 2011). Consistent with a need of IKAP for heart development, RT-PCR analyses showed that expression of the transcription factors *Gata5* and *Smad4*, which are essential for heart morphogenesis, are significantly down-regulated in *Ikbkap* null embryos (Dietrich *et al.*, 2011). Homozygosity for an *Ikbkap* allele lacking exon 20 (*Ikbkap* Δ20; Figure 3) also resulted in a similar phenotype, indicating that the truncated IKAP protein does not retain significant biological function (Dietrich *et al.*, 2011).

From Dietrich *et al.* (2012), by permission of Oxford University Press.

These analyses indicate that inactivation of *Ikbkap* results in several severe developmental abnormalities that are not seen in FD. Also, inactivation of *Ikbkap* in neural crest cell precursors results in cleft secondary palate (Jackson *et al.*, 2014; Dietrich and Dragatsis, unpublished results), a severe developmental malformation that is never observed in FD patients. Keeping in mind that FD has pleiotropic manifestations, and that the IVS20+T > C mutation in FD patients results in reduced levels of *IKBKAP* expression in all tissues, it becomes obvious that *global reduction* and not tissue-specific elimination of *Ikbkap* expression is required to generate a model for FD.

### Mouse models of FD

So far, all attempts to generate a mouse model for FD by introducing the FD major haplotype mutation in the mouse genome have been unsuccessful.

Slaugenhaupt and collaborators generated a series of transgenic mice carrying different copy numbers of a human *IKBKAP* bacterial artificial chromosome (BAC) carrying the FD IVS20+6T > C mutation (FD BAC). Characterization of the splicing pattern of human *IKBKAP* mRNA in several transgenic lines by semi-quantitative RT-PCR revealed that they recapitulated faithfully the same tissue-specific splicing pattern seen in FD patients (Hims *et al.*, 2007). As expected, none of the transgenic lines had FD phenotypic features when normal levels of endogenous mouse IKAP protein was expressed. In an effort to elicit an FD phenotype, FD BAC mice were crossed into an *Ikbkap* null background. Unfortunately, none of the compound FD BAC transgenic/*Ikbkap* null mice recapitulated the FD phenotype. Although the FD BAC mice do not model FD, they can be used to test *in vivo* the efficacy of compounds that have the potential to correct the FD splicing defect (Shetty *et al.*, 2011).

Using another approach, Ast and collaborators generated a mouse *Ikbkap* allele in which exon 20 and its two flanking introns were replaced by the corresponding human FD sequence that contains the IVS20+6T > C mutation (Bochner *et al.*, 2013). Contrary to what was expected, mice homozygous for this mutation were viable and did not exhibit any of the human FD phenotypes. Quantitative RT-PCR analyses revealed that insertion of the FD human mutation resulted in a different tissue-specific splicing pattern in the mouse compared to what is observed in FD patients. In the mouse, significant skipping of exon 20 occurred in the liver, but not in the nervous system. This differential pattern of exon 20 skipping most likely results from differences in the mechanisms of splicing sequence recognition between the two species.

Other research groups also attempted to generate an FD mouse model by introducing the FD point mutation into the mouse *Ikbkap* allele and were equally unsuccessful (D. Brenner personal communication; Dietrich and Dragatsis, unpublished results). Although the reasons for this failure are still unclear, it is possible that the sequence and/or size of the introns surrounding the FD point mutation might be the main reason for the technical difficulties, or that differences between the mouse and human splicing machinery might be the underlying impediment for the success of this approach. Since complete inactivation of *Ikbkap* in mice is embryonic lethal, and FD carriers (heterozygous for the point mutation) expressing 50% the levels of normal full length IKAP protein are asymptomatic, the generation of a successful FD model likely requires that the level of normal IKAP protein be reduced between 0 and 50% the normal levels.

Successful FD mouse models were finally developed with the generation of compound *Ikbkap* ^Δ*20/flox*^ mice (Dietrich *et al.*, 2012). In practical terms, although the compound mouse does not carry the FD major mutation, molecularly the consequence of the generated mutations is the same: expression of an *Ikbkap* mRNA lacking exon 20 from the Δ20 allele and highly reduced wild-type (wt) *Ikbkap* mRNA levels from a hypomorphic Ikbkap floxed allele due to the loxP insertion interference (Figure 3).

**Figure 4:**
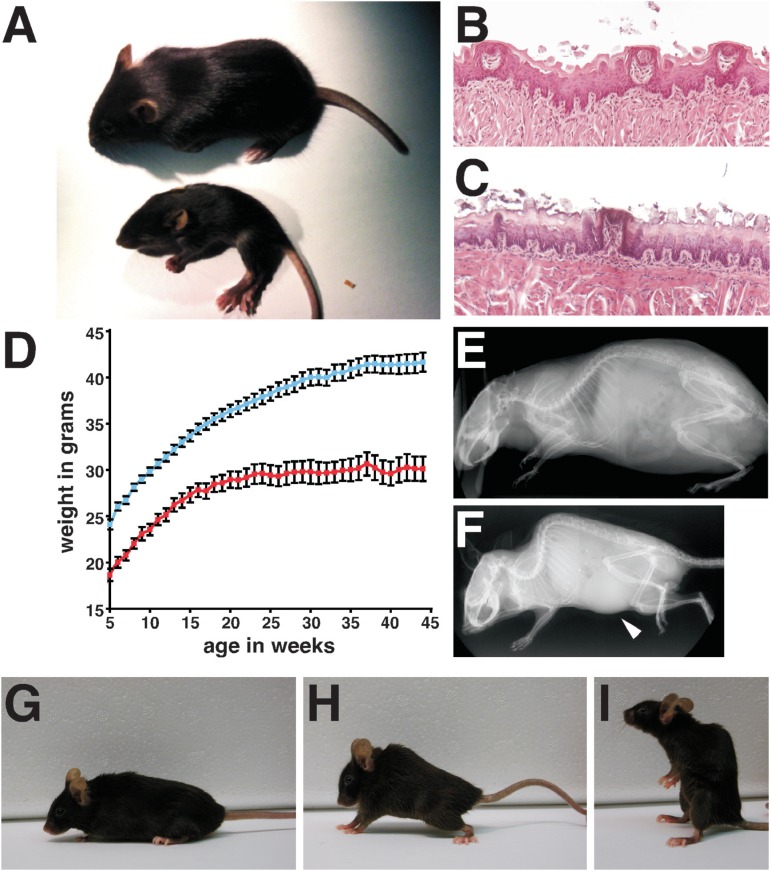
Postnatal characteristics of *Ikbkap* ^Δ*20/flox*^ and *Ikbkap* ^*flox/flox*^ mice. (A) Appearance of *Ikbkap* ^Δ*20/flox*^ mice at P18. WT (top) and *Ikbkap* ^Δ*20/flox*^ (bottom) littermates were photographed side by side. Note that the mutant *Ikbkap* ^Δ*20/flox*^ mouse is significantly smaller than its WT littermate, exhibits abnormal posture, and puffy feet. (B and C) Representative histological examinations of tongue fungiform papillae. Tongues of P18 WT (B) and *Ikbkap* ^Δ*20/flox*^ (C) littermates were processed for paraffin embedding, and coronal sections of the anterior part of the tongue were stained with H&E. Note that the three fugiform papillae of the WT littermate appear normal (B), while in the mutant the fungiform papilla shown is degenerating (C). (D) Postnatal growth curves of control (blue, n=37) and *Ikbkap* ^*flox/flox*^ (red, n=14) male mice. Similar results were found for female mice. Data are represented as Mean ± SEM. (E and F) MicroCT scans of 11-month-old WT (E) and Ikbkap^Δ20/flox^ (F) littermate male mice. Note that the mutant is significantly smaller than control and displays a severe curvature of the spine (kyphosis). In this scan, a significant enlargement of the bladder is also observed in the mutant (arrowhead). (G - I) 16-month-old WT (G), and *Ikbkap* ^*flox/flox*^ (H and I) female littermates. Note the spinal curvature of the mutant (H) and the sitting up posture (I) that it assumes frequently.

Since FD is caused by severe reduction of full-length IKAP protein, it was reasonable to assume that not only the compound *Ikbkap* ^Δ*20/flox*^ mice but also the *Ikbkap* ^*flox/flox*^ mice would represent a model for FD. As it turns out, both *Ikbkap* ^*flox/flox*^ and *Ikbkap* ^Δ*20/flox*^ mice recapitulate several of FD major phenotypic features, including reduced IKAP expression, intrauterine growth retardation, reduced birth weight, failure to thrive, reduced number of fungiform papillae on the tongue, progressive ataxia, kyphosis, stress-induced seizures, decreased temperature perception and impaired development and maintenance of sensory and sympathetic neurons (Figure 4 and Table 1; Dietrich *et al.*, 2012). Similar to FD patients, these FD mouse models also display reduction of expression of genes and proteins involved in myelination in the CNS (Cheishvili *et al.*, 2014a).

From Dietrich *et al.* (2012), by permission of Oxford University Press.

**Table 1:** Ikbkap^Δ20/flox^ and Ikbkap^flox/flox^ mouse models reproduce features of FD.

	FD patients	Ikbkap^Δ20/flox^	Ikbkap^flox/flox^
Reduced expression of full-length IKAP protein	5-20% of controls in CNS	5% of controls in CNS tissues	10% of controls in CNS tissues
Intrauterine growth retardation	+	+	+
Low birth weight	80% of controls	70% of controls	85% of controls
Poor suck, uncoordinated swallow at birth	+	+	+
Poor weight gain	+	+	+
Short stature	+	+	+
Reduced life-span	+	+	+
Dysautonomic crisis	+	N/A	N/A
Seizure susceptibility	+	+	+
Gastrointestinal dysfunction	+	+	+
Absence of overflow emotional tears	+	N/A	N/A
Optic neuropathy	+	+	+
Tongue: Reduced numbers of fungiform papillae	smooth tongue	45% reduction	30% reduction
Decreased deep tendon reflexes	+	N/D	N/D
Muscle spindle abnormalities	Impaired function	N/D	Reduced sensory innervation
Absent axon flare following intradermal histamine injection	+	N/D	N/D
Poor coordination/balance	+	+	+
Spinal abnormalities	+	+	+
Hydronephrosis	+	+	+
Decreased temperature perception	+	+	+
Decreased volume of DRGs	+	45% of controls at birth	70% of controls at birth
Decreased neuronal numbers in DRGs in adults	10-20% of controls	75% reduction in nociceptive neurons	20% reduction in nociceptive neurons
Decreased volume of sympathetic ganglia at birth	N/D	45% of controls at birth	70% of controls at birth
Decreased volume of SCGs in adults	30% of controls	N/D	30% of controls
Decreased neuronal numbers in SCGs in adults	10% of controls	N/D	20% of controls

+ Present;

N/A Not Applicable;

N/D Not Determined.

From Dietrich *et al.* (2012), by permission of Oxford University Press.

Intriguingly, although both *Ikbkap* ^*flox/flox*^ and *Ikbkap* ^Δ*20/flox*^ mouse models recapitulate FD, the severity is strikingly higher in the compound *Ikbkap* ^Δ*20/flox*^ model (Table 1). In addition to a milder disease phenotype, *Ikbkap* ^*flox/flox*^ mice also have a significant increase in longevity. This suggests that a slight increase in IKAP expression is sufficient to greatly ameliorate the symptoms of the disease. Slight differences in IKAP expression (possibly due to differences in splicing efficiency) might therefore also explain the variability in phenotypic expression observed among FD patients. Another important finding through analyses of these models is that intrauterine development of sympathetic ganglia is not extensively compromised even when IKAP levels are as low as 5% of normal levels, and that degeneration and neuronal cell loss in the sympathetic ganglia occurs mostly postnatally. Significantly, as seen in young FD patients, a portion of the remaining neurons in the sympathetic ganglia of postnatal FD mouse models appear small and vacuolated, an indication of dysfunction and ongoing degeneration (Dietrich *et al.*, 2012).

Taken together, these two findings have major implications for therapeutics, since they suggest that even a slight increase in IKAP expression postnatally might be sufficient to halt the progression of sympathetic neuronal loss, and possibly revert other features as well.

Recently, a phenotypic model of FD in which *IKBKAP* mRNA splicing and expression can be modulated was generated by introducing the complete human *IKBKAP* gene (BAC transgene) with the major FD splice mutation (Tg^FD9^; Hims *et al.*, 2007) into the *Ikbkap* ^Δ*20/flox*^ mouse model. The introduction of the human *IKBKAP* FD transgene attenuated the severe FD phenotype observed in the *Ikbkap* ^Δ*20/flox*^ mouse, while still recapitulating FD major features and recreating the same tissue-specific missplicing defect seen in FD patients (Morini *et al.*, 2016). With the availability of mouse models that faithfully recapitulate major features of FD, several questions regarding FD disease process as well as testing of novel therapeutic strategies can now be addressed *in vivo*.

## Conclusions and perspectives

With the identification of the genetic cause of FD, *in vitro* and *in vivo* studies have provided important information related to the normal function of IKAP. In particular, the finding that IKAP plays an essential role in cytoskeleton remodeling and target field innervation provides a plausible explanation for the PNS deficits of the disease. An exciting challenge for the near future is to further elucidate the essential pathways that are disrupted in FD in neuronal and non-neuronal tissues, so that more comprehensive and efficient therapies can be developed and applied to the existing patients. In addition, further understanding of the mechanisms underlying the developmental defects of FD may shed light into other related disorders. For instance, the emerging possible role of IKAP in neurotrophin retrograde transport and signaling - if proven correct - would link the molecular pathways of FD to at least two other HSANs (HSAN IV and HSAN V). With the availability of *in vitro* and *in vivo* models for FD, the answers to these questions are now close at hand.
